# Monocytes and immune evasion strategies in HIV: Unraveling the intricacies

**DOI:** 10.1097/MD.0000000000046476

**Published:** 2026-05-12

**Authors:** Emmanuel Ifeanyi Obeagu, Getrude Uzoma Obeagu

**Affiliations:** a Department of Biomedical and Laboratory Science, Africa University, Mutare, Zimbabwe; b School of Nursing Science, Kampala International University, Ishaka, Uganda.

**Keywords:** chronic immune activation, HIV, immune evasion, immune modulation, monocytes, therapeutic strategies, viral reservoirs

## Abstract

Human immunodeficiency virus (HIV) employs sophisticated immune evasion strategies to establish persistent infection within the host. Monocytes, crucial components of the innate immune system, play a pivotal role in shaping the immune response against HIV. This review delves into the intricate interplay between monocytes and HIV, focusing on the virus’s strategies to evade immune surveillance and the implications for disease progression. The phenotypic diversity of monocyte subsets, including classical, intermediate, and non-classical monocytes, adds complexity to their roles in HIV pathogenesis. The virus exploits monocytes as both targets for infection and reservoirs, impacting viral dissemination and persistence. Understanding the mechanisms of monocyte–HIV interactions is crucial for unraveling the dynamics of immune evasion. HIV employs various immune evasion strategies that directly manipulate monocyte functions. These include viral entry mechanisms that exploit monocyte receptors, modulation of monocyte cytokine responses, and interference with antigen presentation. The virus also shapes the monocyte phenotype to establish a pro-inflammatory environment that fosters viral replication while evading immune detection.

## 1. Introduction

Human immunodeficiency virus (HIV) continues to pose a significant global health challenge, with persistent efforts directed towards understanding the complexities of viral pathogenesis and developing effective therapeutic interventions.^[1]^ A critical aspect of HIV infection involves the virus’s adept ability to evade the host immune system, allowing for prolonged viral persistence and disease progression.^[2]^ Monocytes, key players in the innate immune response, play a central role in shaping the host’s defense mechanisms against pathogens, including HIV.^[3]^ The interplay between monocytes and HIV is a dynamic and multifaceted relationship, with monocytes serving both as targets for viral infection and as critical contributors to immune responses against the virus.^[4]^ This review aims to explore the intricate mechanisms employed by HIV to evade immune detection within the monocyte population, shedding light on the implications for viral persistence, chronic immune activation, and the challenges in developing curative strategies. Understanding the phenotypic and functional diversity of monocyte subsets, including classical, intermediate, and non-classical monocytes, provides a foundation for unraveling their distinct roles in the context of HIV infection.^[5]^ The virus exploits monocytes not only as susceptible cells for infection but also as reservoirs that contribute to the establishment of viral sanctuaries within the host. This dual role of monocytes highlights their significance in HIV pathogenesis and the potential hurdles in achieving complete viral eradication.

Understanding the role of monocytes in the context of immune evasion strategies employed by HIV sheds light on the intricate dynamics of viral pathogenesis and host defense mechanisms. Monocytes, a type of white blood cell, serve as crucial players in the immune system, functioning both as antigen-presenting cells and as precursors to tissue macrophages and dendritic cells. Their ability to migrate to sites of inflammation and infection positions them as key regulators of immune responses. In the context of HIV infection, monocytes play a multifaceted role, being not only targets for viral replication but also serving as vehicles for viral dissemination throughout the body. One of the most intriguing aspects of HIV pathogenesis is its ability to evade host immune surveillance, perpetuating chronic infection. HIV employs various strategies to evade immune detection and elimination, many of which involve subversion of monocyte function. For instance, HIV can exploit the migratory capacity of monocytes to disseminate to distant tissues, establishing reservoirs of latent infection. Additionally, HIV can modulate monocyte phenotype and function, impairing their ability to mount effective antiviral responses and promoting a pro-inflammatory environment conducive to viral persistence.^[3,4]^

Moreover, HIV has been shown to directly infect and replicate within monocytes, further complicating the interplay between the virus and the immune system. Infected monocytes can serve as long-lived reservoirs of HIV, contributing to viral persistence despite antiretroviral therapy. Furthermore, HIV-induced alterations in monocyte gene expression and cytokine secretion can have profound effects on immune homeostasis, exacerbating systemic inflammation and immune dysfunction observed in HIV-infected individuals. Understanding the intricate interplay between monocytes and HIV is crucial for developing novel therapeutic strategies aimed at combating viral persistence and restoring immune function in HIV-infected individuals. Targeting monocyte-mediated pathways of viral dissemination and immune evasion holds promise for enhancing the efficacy of current antiretroviral therapies and ultimately achieving a functional cure for HIV. By unraveling the complexities of monocyte–HIV interactions, we can pave the way towards more effective treatments that address the underlying mechanisms driving chronic HIV infection and associated immunopathology.^[5–7]^

## 2. Aim

The aim of exploring the intricate relationship between monocytes and immune evasion strategies in HIV is to unravel the underlying mechanisms driving viral persistence and chronic infection. By understanding how HIV manipulates monocyte function and phenotype to evade immune detection and elimination, we can identify novel therapeutic targets for combating viral dissemination and restoring immune homeostasis.

### 2.1. Ethical approval

Not applicable.

### 2.2. Distinction between 2 different HIV viruses: HIV-1 and HIV-2

HIV-1 and HIV-2 are 2 distinct strains of the HIV that cause acquired immunodeficiency syndrome (AIDS).^[1]^ While they share similarities in terms of their mode of transmission and pathogenesis, there are several key differences between the 2 viruses:

### 2.3. Genetic variation

HIV-1 is more prevalent globally and exhibits greater genetic diversity compared to HIV-2. There are multiple subtypes (clades) of HIV-1, which are distributed geographically. HIV-2 is less prevalent and primarily found in West Africa, although cases have been reported in other regions. It is genetically distinct from HIV-1 and is divided into subtypes A through H.^[2]^

### 2.4. Transmission

HIV-1 is transmitted more efficiently than HIV-2 and is the predominant cause of the global HIV/AIDS pandemic. It is primarily transmitted through sexual contact, exposure to infected blood or blood products, and perinatal transmission from mother to child. HIV-2 is less efficiently transmitted through sexual contact compared to HIV-1. It is also transmitted through exposure to infected blood or blood products and from mother to child, but it has a lower perinatal transmission rate than HIV-1.^[3]^

### 2.5. Pathogenicity

HIV-1 infection progresses more rapidly to AIDS and is associated with higher viral loads and greater CD4 + T cell depletion compared to HIV-2. HIV-2 infection typically progresses more slowly to AIDS and is associated with lower viral loads and a slower decline in CD4 + T cell counts compared to HIV-1. Individuals infected with HIV-2 may have a longer asymptomatic phase and a lower risk of developing AIDS-related complications.^[4]^

### 2.6. Antiretroviral therapy (ART) response

Most antiretroviral drugs used for the treatment of HIV/AIDS are more effective against HIV-1 than HIV-2. However, some drugs, such as certain protease inhibitors, have activity against both HIV-1 and HIV-2. HIV-2 is inherently less susceptible to certain antiretroviral drugs compared to HIV-1, which may impact treatment outcomes. Different drug regimens and monitoring strategies may be required for HIV-2-infected individuals.^[5]^

### 2.7. Viral load testing

Commercially available viral load assays are designed primarily to quantify HIV-1 RNA levels in plasma. These assays may not accurately measure HIV-2 viral load. Specific viral load assays are required to accurately quantify HIV-2 RNA levels in plasma, as standard HIV-1 assays may underestimate HIV-2 viral load.^[6]^

### 2.8. Review methodology search strategy

PubMed, Embase, and Web of Science databases were systematically searched for relevant articles published between 1990 and 2022. Search terms included “monocytes,” “HIV,” “immune evasion,” “viral persistence,” “pathogenesis,” and related terms.

### 2.9. Inclusion criteria

Studies investigating the interaction between monocytes and HIV, immune evasion strategies employed by HIV, and their implications for viral pathogenesis.

### 2.10. Exclusion criteria

Studies not directly relevant to the topic, such as those focused solely on other immune cells or unrelated pathogens.

### 2.11. Study selection

Two independent reviewers screened titles and abstracts of retrieved articles to assess eligibility for full-text review. Full-text articles were assessed for eligibility based on inclusion and exclusion criteria. Any discrepancies between reviewers were resolved through discussion and consensus.

### 2.12. Data extraction

Data were extracted from eligible studies using a standardized data extraction form. Extracted data included study characteristics (e.g., author, year, study design), key findings related to monocyte–HIV interactions, immune evasion strategies, and implications for viral persistence and immune dysfunction.

### 2.13. Data synthesis

Data from eligible studies were synthesized narratively to provide a comprehensive overview of the relationship between monocytes and HIV immune evasion strategies. Key findings were organized into thematic sections, including monocyte subsets and functions, mechanisms of immune evasion, viral dissemination, impact on immune responses, and therapeutic targets. Tables were used to summarize key data and facilitate comparison between studies.

### 2.14. Quality assessment

The methodological quality of included studies was assessed using appropriate tools (e.g., Newcastle–Ottawa Scale for cohort studies, Cochrane Risk of Bias tool for randomized controlled trials). Quality assessment was performed independently by 2 reviewers, with any discrepancies resolved through discussion.

### 2.15. Monocyte susceptibility to HIV infection

HIV infection continues to pose a global health challenge, with approximately 38 million people living with the virus worldwide. Despite significant progress in understanding the pathogenesis of HIV, the intricate interactions between the virus and host immune cells, particularly monocytes, remain a subject of intense investigation.^[8–12]^ Monocytes, as key players in the innate immune system, serve as sentinel cells involved in pathogen recognition, phagocytosis, and immune regulation.^[13]^ Emerging evidence suggests that monocytes play a crucial role in HIV pathogenesis, serving as both targets for viral entry and reservoirs for persistent infection.^[14]^ This review aims to shed light on the susceptibility of monocytes to HIV infection, unraveling the mechanisms underlying viral entry, replication, and the establishment of reservoirs within these cells. Monocyte subsets, characterized by distinct surface markers and functional properties, exhibit differential susceptibility to HIV.^[15]^ The classical (CD14++CD16-), intermediate (CD14++CD16+), and non-classical (CD14 + CD16++) monocyte subsets display unique responses to viral exposure, influencing the dynamics of infection. Understanding the susceptibility of these subsets is essential for comprehending the heterogeneity in viral replication, immune activation, and disease progression observed in HIV-infected individuals.

### 2.16. Viral persistence in monocytes

HIV has long been recognized for its ability to exploit various immune cells, and monocytes, as crucial components of the innate immune system, are no exception.^[16]^ Emerging evidence suggests that different monocyte subsets exhibit distinct levels of susceptibility to HIV. The classical (CD14++CD16-), intermediate (CD14++CD16+), and non-classical (CD14 + CD16++) subsets display varying responses to viral exposure, influencing the kinetics of infection and subsequent immune activation.^[15]^ Monocytes, particularly the classical subset, express high levels of CD4 and CCR5 receptors, rendering them susceptible to R5-tropic HIV strains. The interaction between viral glycoproteins and these receptors initiates the entry process.^[16]^ In certain contexts, CXCR4 co-receptor expression on monocytes may facilitate infection by X4-tropic HIV strains, broadening the spectrum of susceptible cells.^[17]^ Monocytes express pattern recognition receptors: such as Toll-like receptors that recognize viral components.^[18]^ The activation of these receptors upon HIV exposure triggers signaling cascades, influencing the cellular milieu and potentially modulating susceptibility. Type I interferons induced by viral sensing can exert both pro- and antiviral effects in monocytes, impacting the outcome of infection. Following entry, HIV undergoes reverse transcription within the monocyte cytoplasm, leading to the formation of viral DNA. The integrated viral DNA can actively transcribe viral RNA, leading to the production of new virions within the monocyte. Monocytes, with their relatively long lifespan and ability to traffic to various tissues, serve as potential reservoirs for latent and persistent HIV.

### 2.17. Immune modulation by monocytes

Monocytes, as central components of the innate immune system, play a pivotal role not only in the initial response to pathogens but also in shaping the overall immune milieu. This section explores the diverse ways in which monocytes modulate the immune response during HIV infection, impacting both innate and adaptive components of immunity. Upon encountering HIV, monocytes can release a spectrum of pro-inflammatory cytokines such as interleukin-1β, interleukin-6, and tumor necrosis factor-α. These cytokines contribute to the overall inflammatory environment associated with chronic immune activation in HIV infection.^[19]^ Monocytes are key producers of chemokines, influencing the recruitment and activation of other immune cells. The dysregulated release of chemokines by monocytes contributes to immune dysregulation and may influence disease progression.

Monocytes, particularly the CD16 + subset, can function as antigen-presenting cells in HIV infection. They capture viral antigens, process them, and present peptides to CD4 + T cells, initiating adaptive immune responses. Monocyte-derived antigen-presenting cells can activate CD4 + T cells, contributing to the expansion of virus-specific responses. However, chronic immune activation may also lead to T cell exhaustion and dysfunction.^[3]^ Monocytes can express immune checkpoint molecules, such as PD-L1, which may modulate T cell responses.^[20]^ The interaction between monocytes and T cells via checkpoint molecules contributes to the regulation of immune activation and potentially influences HIV-specific immune responses. Activated monocytes can traffic to inflamed tissues, modulating the local immune microenvironment. This trafficking contributes to the recruitment of immune cells and may influence the dynamics of viral replication and persistence. Monocytes can interact with stromal cells within tissues, influencing fibrosis and tissue integrity. These interactions may contribute to HIV-associated comorbidities, such as cardiovascular complications. Monocytes can influence B cell responses through cytokine production and direct interactions. The modulation of antibody responses may have implications for HIV vaccine development and humoral immunity. Certain monocyte subsets, particularly the CD16 + subset, can produce immunosuppressive cytokines such as interleukin-10 (IL-10). Elevated IL-10 levels contribute to immune dysfunction and may facilitate viral persistence.^[21]^

### 2.18. Relationship between HIV and immune evasion

The relationship between HIV and immune evasion is complex and multifaceted, involving a series of strategies employed by the virus to evade detection and elimination by the host immune system. HIV exhibits high genetic variability and rapid mutation rates, leading to the emergence of diverse viral strains within an infected individual. This genetic diversity allows HIV to continuously evade immune recognition and neutralization by the host antibodies. HIV can alter its cellular tropism, enabling it to infect and replicate within various immune cell types, including CD4 + T cells, macrophages, and dendritic cells. By infecting different cell types, HIV can evade immune responses targeting specific cell populations. HIV employs various strategies to evade immune detection and elimination. These include downregulating expression of viral antigens on the surface of infected cells, shielding viral proteins from recognition by host antibodies, and suppressing antiviral immune responses through the release of immunosuppressive cytokines. HIV can establish latent infections in long-lived reservoirs, such as resting memory CD4 + T cells and tissue macrophages. Latently infected cells do not produce viral proteins or undergo cytopathic effects, making them invisible to the immune system and resistant to antiretroviral therapy. Chronic HIV infection leads to dysregulation of the immune system, characterized by persistent immune activation, exhaustion of immune effector cells, and loss of immune function. This immune dysregulation contributes to disease progression and impairs the host’s ability to control viral replication. HIV can evade immune recognition by acquiring mutations in viral epitopes targeted by host immune responses. These escape mutations enable the virus to evade neutralization by antibodies and cytotoxic T lymphocytes, leading to immune evasion and persistence of infection. HIV can modulate host immune responses to its advantage. For example, the virus induces chronic inflammation, which creates a pro-inflammatory environment that promotes viral replication and pathogenesis while impairing immune function. HIV can interfere with antigen presentation pathways, impairing the ability of infected cells to present viral antigens to cytotoxic T cells and evade immune recognition.^[3,20,21]^

### 2.19. Monocytes in HIV-induced inflammation

HIV infection is characterized by chronic immune activation and inflammation, contributing significantly to disease progression and the development of comorbidities. Monocytes, as key players in the innate immune system, play a central role in orchestrating and perpetuating the inflammatory response during HIV infection.^[22]^ Monocytes are potent producers of pro-inflammatory cytokines, including interleukin-1β, interleukin-6, and tumor necrosis factor-α. Elevated levels of these cytokines are observed in HIV-infected individuals and contribute to systemic inflammation.^[20]^ Monocytes release cytokines that can activate other immune cells, creating a pro-inflammatory feedback loop. This sustained signaling contributes to chronic immune activation, a hallmark of HIV infection.^[3]^

Monocytes produce chemokines such as CCL2, CCL3, and CCL5, influencing the recruitment of immune cells to sites of infection and inflammation.^[22]^ The dysregulated release of chemokines contributes to the accumulation of immune cells in tissues and systemic inflammation.^[23]^ Activated monocytes can migrate to inflamed tissues, further amplifying the local immune response and contributing to tissue-specific pathologies associated with HIV-induced inflammation.^[24]^ Monocytes, upon activation, upregulate adhesion molecules such as ICAM-1 and VCAM-1. The interaction between activated monocytes and endothelial cells contributes to endothelial activation and vascular dysfunction.^[25]^ HIV-induced inflammation, mediated in part by monocytes, contributes to a pro-thrombotic state, increasing the risk of cardiovascular complications in infected individuals. Monocytes, through the release of cytokines and direct interactions, can modulate T cell responses. While initially contributing to antiviral immunity, chronic immune activation may lead to T cell dysfunction and exhaustion. Monocytes influence B cell responses, potentially impacting antibody production and the effectiveness of humoral immunity.

Monocyte-mediated inflammation is implicated in the development of cardiovascular diseases in HIV-infected individuals.^[26]^ The interplay between activated monocytes and the vascular endothelium contributes to atherosclerosis and increased cardiovascular risk. In the central nervous system, monocytes contribute to neuroinflammation and may play a role in HIV-associated neurocognitive disorders. Targeting monocyte-mediated inflammation represents a potential therapeutic approach to mitigate the detrimental effects of chronic immune activation in HIV.^[27]^ Strategies aimed at modulating monocyte–endothelial interactions may have implications for reducing the risk of cardiovascular complications in HIV-infected individuals. Table 1 shows monocyte subsets and their characteristics in HIV.

**Table 1:** Monocyte subsets and functions in HIV infection.

Monocyte subset	Function in HIV infection
Classical	Phagocytosis, antigen presentation.
Intermediate	Pro-inflammatory cytokine production.
Non-classical	Tissue repair, surveillance.
HIV target	Direct viral replication, dissemination.

### 2.20. Immune checkpoints and monocyte dysfunction

Immune checkpoints play a crucial role in regulating immune responses and maintaining self-tolerance.^[28]^ In the context of HIV infection, dysregulation of immune checkpoint molecules on monocytes contributes to immune dysfunction and may impact the overall immune landscape. Programmed cell death protein 1 (PD-1) and its ligand PD-L1 are expressed on monocytes during HIV infection.^[29]^ PD-1 expression on monocytes may contribute to dysfunction, impairing their ability to mount effective antiviral responses. Interactions between PD-1 on monocytes and its ligands on T cells can lead to T cell exhaustion, limiting the effectiveness of adaptive immune responses against HIV.^[30]^ PD-L1 on monocytes may negatively regulate inflammatory responses, contributing to a state of immune suppression and chronic inflammation. Cytotoxic T-lymphocyte-associated protein 4 (CTLA-4) expression is induced on monocytes during HIV infection. CTLA-4 engagement may modulate monocyte functions and influence the overall immune response. Interaction between CTLA-4 on monocytes and CD80/CD86 on antigen-presenting cells can lead to T cell inhibition, impacting the magnitude of adaptive immune responses against HIV. CTLA-4 on monocytes may interfere with co-stimulatory signals required for optimal T cell activation, contributing to immunosuppression.

T cell immunoglobulin and mucin domain 3 and its ligand galectin-9 are upregulated on activated monocytes during HIV infection.^[31]^ Interaction between T cell immunoglobulin and mucin domain 3 and galectin-9 on monocytes may induce dysfunction, impairing their ability to regulate immune responses and contribute to the establishment of an immunosuppressive environment. Immune checkpoint molecules on monocytes can modulate inflammatory signaling pathways, influencing the release of cytokines and chemokines. Dysregulated expression of immune checkpoints may contribute to a feedback loop that perpetuates chronic immune activation and inflammation in HIV-infected individuals. Therapeutic strategies involving checkpoint inhibitors are being explored to alleviate immune dysfunction in HIV.^[32]^ However, caution is warranted to balance immune activation and avoid excessive inflammation. Combinatorial approaches targeting both viral replication and immune dysfunction, including immune checkpoint blockade, may offer synergistic benefits in restoring immune homeostasis. Table 2 shows immune checkpoints and monocyte dysfunction in HIV.

**Table 2:** HIV evasion strategies targeting monocytes.

Evasion strategy	Mechanism
Latency establishment	Integration into monocyte genome, hiding from immune surveillance.
Immune dysregulation	Induction of pro-inflammatory cytokines, suppression of antiviral responses.
Tropism alteration	Adapting to infect and replicate within monocytes.
Immune cell modulation	Manipulation of monocyte phenotype and function.

HIV = human immunodeficiency virus.

### 2.21. Monocyte trafficking and viral dissemination

Monocytes, as key components of the innate immune system, play a critical role in the dissemination of HIV throughout the body.^[33]^ Their ability to migrate to various tissues and organs, coupled with their susceptibility to HIV infection, positions monocytes as crucial contributors to viral spread. Activated monocytes respond to chemotactic signals, such as chemokines, guiding their migration to inflamed tissues and sites of infection. Monocytes, upon reaching inflamed tissues, contribute to the amplification of local immune responses by releasing pro-inflammatory cytokines and interacting with other immune cells. Monocytes serve as circulating viral reservoirs, carrying infectious HIV particles throughout the bloodstream.^[34]^ This contributes to the systemic nature of HIV infection and facilitates viral dissemination to distant anatomical sites.

The migration of infected monocytes to specific tissues may influence the establishment of viral reservoirs in different anatomical compartments, including the central nervous system and lymphoid organs. Monocytes can transmigrate across endothelial barriers to enter tissues, a process facilitated by adhesion molecules and chemokine gradients. The interaction between activated monocytes and endothelial cells can lead to the disruption of endothelial integrity, contributing to inflammation and potentially facilitating viral entry into tissues. Infected monocytes have the potential to transfer virions to CD4 + T cells through a process known as trans-infection. This mechanism amplifies the spread of the virus within the host. Monocytes, once infiltrated into tissues, may modulate the local immune microenvironment. This tissue-specific response can impact the dynamics of viral replication and contribute to the establishment of viral reservoirs.^[30]^ Monocytes, as long-lived cells, can harbor latent HIV, posing challenges for antiretroviral therapy.^[4]^ The persistence of viral reservoirs within monocytes contributes to the difficulty in achieving complete viral eradication. Strategies aimed at modulating monocyte migration and tissue infiltration may impact the establishment of viral reservoirs and limit viral dissemination. Integrating therapeutic interventions targeting both viral replication and monocyte trafficking may offer a comprehensive strategy to curtail HIV dissemination.

### 2.22. Modulation of immune processes by the different HIV proteins

HIV employs various proteins to modulate immune processes, allowing the virus to evade detection and elimination by the host immune system. Here’s an overview of how different HIV proteins contribute to immune modulation:

### 2.23. Envelope glycoprotein (Env)

Env facilitates viral entry into target cells by binding to CD4 receptors and co-receptors (e.g., CCR5 or CXCR4) on the surface of host cells. Env can also induce syncytium formation, leading to the fusion of infected and uninfected cells, thereby promoting viral spread and immune evasion.^[30]^

### 2.24. Accessory proteins (Vpr, Vif, Vpu, and Nef)

Vpr protein has been shown to induce cell cycle arrest and apoptosis in infected CD4 + T cells and macrophages, thereby promoting viral persistence and immune evasion. Vif protein counteracts the host restriction factor APOBEC3G, which inhibits viral replication by inducing hypermutation of the viral genome. By neutralizing APOBEC3G, Vif facilitates viral replication and immune evasion. Vpu protein promotes the degradation of CD4 receptors on the surface of infected cells, thereby preventing their recognition by cytotoxic T cells and promoting viral escape from immune surveillance. Nef protein downregulates expression of major histocompatibility complex class I molecules on the surface of infected cells, reducing their recognition by cytotoxic T cells. Nef also enhances viral infectivity and replication by modulating host cell signaling pathways.^[30,31]^

### 2.25. Transactivator of transcription (Tat)

Tat protein enhances viral transcription by binding to the viral long terminal repeat promoter region, leading to increased production of viral RNA and proteins. Tat can also dysregulate host immune responses by promoting the secretion of pro-inflammatory cytokines and chemokines, contributing to chronic immune activation and inflammation.^[32]^

### 2.26. Matrix protein (MA)

MA protein plays a role in viral assembly and release from infected cells. MA can also modulate host cell signaling pathways and immune responses, although its specific immunomodulatory functions are not fully understood.^[33]^

### 2.27. Reverse transcriptase (RT) and integrase (IN)

RT and IN are essential for viral replication and integration of viral DNA into the host genome. While RT and IN primarily function in viral replication, they may indirectly modulate host immune responses through their interactions with host cell factors and signaling pathways.^[34]^

### 2.28. Therapeutic interventions

The complex interplay between monocytes and HIV infection has prompted the exploration of therapeutic interventions aimed at modulating monocyte responses to mitigate viral replication, inflammation, and the establishment of reservoirs.^[35]^ The cornerstone of HIV treatment is ART, which inhibits viral replication and reduces viral load. Early initiation of ART can limit the establishment of viral reservoirs within monocytes and other immune cells. Immune checkpoint inhibitors, such as anti-PD-1 or anti-CTLA-4 antibodies, are being investigated to enhance antiviral immune responses and alleviate monocyte dysfunction.^[36]^ Targeting monocyte-mediated inflammation with immunomodulatory agents may help restore immune homeostasis and reduce chronic immune activation.

Latency reversal agents aim to activate latent HIV within monocyte reservoirs, rendering the virus susceptible to immune clearance or antiretroviral drugs.^[37]^ Identifying safe and effective latency reversal agents specific to monocytes is an ongoing challenge, and careful consideration is required to prevent excessive inflammation. Targeting chemokine receptors involved in monocyte trafficking may disrupt their migration to inflamed tissues and reduce the dissemination of HIV.^[38]^ Drugs targeting CCR5, a co-receptor for HIV entry, can inhibit viral entry into monocytes and potentially limit the establishment of reservoirs. Modulating inflammatory responses with anti-inflammatory agents may help mitigate monocyte-mediated inflammation and reduce the risk of associated comorbidities. Considering the link between monocyte-mediated inflammation and cardiovascular complications, interventions targeting cardiovascular risk factors may have therapeutic implications. Combining antiretroviral drugs with immunomodulatory agents, latency reversal agents, or anti-inflammatory approaches may offer synergistic benefits in controlling HIV replication and reducing immune dysfunction.^[39]^ Tailoring therapeutic interventions based on individual patient profiles and viral dynamics may enhance treatment efficacy. Developing vaccines that stimulate robust and durable immune responses against HIV may prevent initial infection of monocytes and limit viral dissemination.

### 2.29. Co-infections and immune evasion

HIV infection is often complicated by the presence of co-infections, which can interact with the virus and modulate immune responses.^[40]^ Co-infections, such as viral, bacterial, or parasitic infections, can alter the immune microenvironment, influencing the activation status of immune cells, including monocytes. The presence of co-infections may either enhance or suppress immune responses, creating a milieu that facilitates or impedes HIV replication and persistence. Co-infecting viruses may exhibit synergistic effects with HIV, enhancing viral replication and potentially accelerating disease progression.^[41]^ In some cases, co-infections may compete with HIV for host resources, leading to a dampened viral replication. This competition may influence the overall viral load and immune activation.

Co-infections may induce changes in monocyte subsets, altering their phenotype and function. This altered phenotype can impact the susceptibility of monocytes to both HIV and co-infecting agents. The expression of co-receptors and activation markers on monocytes may be modulated by co-infections, influencing their ability to interact with and respond to HIV.^[41]^ Co-infections can contribute to heightened immune activation, leading to chronic inflammation. This persistent inflammation may exacerbate immune dysregulation in HIV-infected individuals. Some co-infections may induce immunosuppressive mechanisms, further compromising the host’s ability to mount effective antiviral responses.^[40]^ Certain co-infecting agents, particularly those with the ability to establish latent or persistent infections, may contribute to the pool of viral reservoirs within monocytes and other immune cells. Co-infections may pose challenges for antiretroviral therapy by influencing the persistence of viral reservoirs and complicating the achievement of viral eradication. Integrated therapeutic approaches that consider both HIV and co-infections are essential for effective disease management. Therapies targeting monocytes and immune responses may need to be adapted based on the specific co-infection profile, considering the diverse effects on the immune system. Developing vaccines against prevalent co-infecting agents may contribute to preventing additional infections and reducing the complexity of HIV disease management.^[42]^ In some cases, antimicrobial prophylaxis for common co-infections may be considered to reduce the burden of additional infections and their impact on immune function. Table 1 shows monocyte subsets and functions in HIV infection, Table 2 shows HIV evasion strategies targeting monocytes, Table 3 shows monocyte-mediated HIV dissemination, Table 4 shows impact of HIV on monocyte phenotype, Table 5 shows therapeutic targets for HIV treatment and Table 6 shows consequences of monocyte infection with HIV (provided by the authors).

**Table 3:** Monocyte-mediated HIV dissemination.

Mode of dissemination	Description
Bloodstream	Circulation of infected monocytes throughout the body.
Tissue migration	Infiltration of tissues with infected monocytes.
Viral transmission	Direct transfer of virus from monocytes to target cells.
Reservoir formation	Establishment of long-lived reservoirs of latent infection.

HIV = human immunodeficiency virus.

**Table 4:** Impact of HIV on monocyte phenotype.

Outcome	Description
Viral dissemination	Spreading infection to distant tissues and organs.
Immune dysregulation	Amplification of systemic inflammation.
Reservoir formation	Establishment of long-lived viral reservoirs.
Chronic inflammation	Persistent immune activation and tissue damage.

HIV = human immunodeficiency virus.

**Table 5:** Therapeutic targets for HIV treatment.

Target	Description
Latency reversal agents	Reactivation of latent virus in infected monocytes.
Immune modulators	Restoration of immune function and homeostasis.
Entry inhibitors	Blockade of viral entry into monocytes and other cells.
Reservoir disruptors	Strategies to eliminate or reduce viral reservoirs.

HIV = human immunodeficiency virus.

**Table 6:** Consequences of monocyte infection with HIV.

Outcome	Description
Viral dissemination	Spreading infection to distant tissues and organs.
Immune dysregulation	Amplification of systemic inflammation.
Reservoir formation	Establishment of long-lived viral reservoirs.
Chronic inflammation	Persistent immune activation and tissue damage.

HIV = human immunodeficiency virus.

## 3. Conclusion

The dynamic interplay between monocytes and HIV unfolds as a multifaceted relationship with profound implications for viral pathogenesis, immune responses, and therapeutic strategies. Monocytes, as versatile components of the innate immune system, contribute to the intricate landscape of HIV infection through their susceptibility to viral entry, role in viral dissemination, modulation of immune responses, and establishment of reservoirs. The differential susceptibility of monocyte subsets to HIV, coupled with their ability to serve as viral reservoirs, highlights the complexity of the virus–host interaction. The classical, intermediate, and non-classical monocyte subsets exhibit distinct functions and responses to HIV, influencing the dynamics of viral replication, immune activation, and disease progression.

Immune evasion strategies employed by HIV, including modulation of monocyte functions, further complicate the host–pathogen relationship. The virus exploits monocytes not only as vehicles for dissemination but also as contributors to chronic inflammation, immune dysregulation, and the establishment of long-lived viral reservoirs. Understanding these strategies is paramount for developing targeted therapeutic interventions that address the multifaceted challenges posed by HIV. The impact of monocytes extends beyond their direct interactions with HIV, as co-infections further shape the immune landscape. The presence of additional pathogens can alter monocyte phenotypes, affect viral replication, and contribute to immune dysregulation. Recognizing the interconnectedness of monocytes, HIV, and co-infections underscores the importance of integrated approaches in clinical management and therapeutic interventions.

## Author contributions

**Conceptualization**: Emmanuel Ifeanyi Obeagu.

**Methodology**: Emmanuel Ifeanyi Obeagu, Getrude Uzoma Obeagu.

**Supervision**: Emmanuel Ifeanyi Obeagu.

**Validation**: Emmanuel Ifeanyi Obeagu.

**Visualization**: Emmanuel Ifeanyi Obeagu.

**Writing – original draft**: Emmanuel Ifeanyi Obeagu, Getrude Uzoma Obeagu.

**Writing – review & editing**: Emmanuel Ifeanyi Obeagu, Getrude Uzoma Obeagu.
